# Global shortfalls of knowledge on anuran tadpoles

**DOI:** 10.1038/s44185-023-00027-1

**Published:** 2023-10-30

**Authors:** Florencia Vera Candioti, Diego Baldo, Stéphane Grosjean, Martín O. Pereyra, Javier Nori

**Affiliations:** 1 Unidad Ejecutora Lillo (Consejo Nacional de Investigaciones Científicas y Técnicas–Fundación Miguel Lillo), 4000 San Miguel de Tucumán, Argentina; 2 Laboratorio de Genética Evolutiva “Claudio Juan Bidau”, Instituto de Biología Subtropical (CONICET–Universidad Nacional de Misiones) and Facultad de Ciencias Exactas Químicas y Naturales (UNaM), 3300 Posadas, Argentina; 3 Direction Générale Déléguée aux Collections, Muséum national d’Histoire naturelle, 75005 Paris, France; 4 Instituto de Diversidad y Ecología Animal (CONICET) and Centro de Zoología Aplicada (Universidad Nacional de Córdoba), 5000 Córdoba, Argentina

**Keywords:** Herpetology, Biodiversity

## Abstract

Despite the amount of data on different aspects of biodiversity, such as species distributions, taxonomy, or phylogenetics, there are still significant gaps and biases in the available information. This is particularly true for life history traits, with fragmentary data for most taxa, especially those with complex life cycles. Anurans (frogs and toads) show larval (premetamorphic) stages that are in general radically decoupled from adult forms in most biological aspects. Our understanding of this group is highly uneven, as the main wide-scope investigations focus on adult specimens and larval stages remain unknown for a significant part of the anuran tree. The main purpose of this work was to estimate the extent of knowledge gaps regarding the diversity of tadpoles, interpret their biological and geographical patterns, and discuss possible explanations and implications for other large-scale analyses. Our findings show that more than half of the anuran species described to date still lack information on their embryonic/larval stages. Furthermore, knowledge varies among taxonomic groups, larval ecomorphological guilds, and world ecoregions. Description percentages generally decrease in lineages with a higher proportion of species known or suspected to have endotrophic development. Also, geographic areas with the highest levels of ignorance in larval biology (Tropical Andes and New Guinea) coincide with the highest diversity of endotrophic guilds. Among exotrophic larvae, generalized lentic-lotic tadpoles have the widest distribution and levels of knowledge, whereas specialized lotic, fossorial, and terrestrial forms are more taxonomically and geographically restricted. Further large-scale analyses on tadpole biology are crucial for their impact in varied scientific disciplines including anuran conservation. At a conceptual level, the discussion of the anuran biphasic life cycle is pertinent in the context of shortfalls of biodiversity knowledge and their interrelationships.

## Introduction

Achieving a comprehensive understanding of biodiversity is crucial for advancing applied science^1^. Today, in the BigData era, the availability of information about different aspects of biodiversity—such as species distributions, taxonomy, or phylogenetics—is astonishing. However, there are still significant gaps and biases in the available information often hindering accurate applications^2^. Furthermore, several essential aspects of biodiversity still need to be studied, with scarce or nonexistent information available^3^. This lack of understanding is particularly true for life history traits, with fragmentary data for most taxa but especially those with complex life cycles. The lack of information about the ontogeny of certain organisms, which often involves a complete knowledge gap regarding particular life stages, significantly impacts on our understanding of their general biology and interactions with the environment, hindering our ability to manage or protect these taxa. While this fact has been previously introduced^4^, it still needs to be quantified across groups^5^. A textbook example in this regard is anurans (frogs and toads), whose larval (premetamorphic) stages are in general radically decoupled from adult forms in most biological aspects^6^. Both life stages may face independent threats from human activities, and survival may depend on completing each stage successfully^7^. Regrettably, our understanding of this group is highly uneven, as primary wide-scope research focuses on the adult phase^8,9^, and larval stages still need to be discovered for a significant part of the anuran tree.

**Fig. 1:**
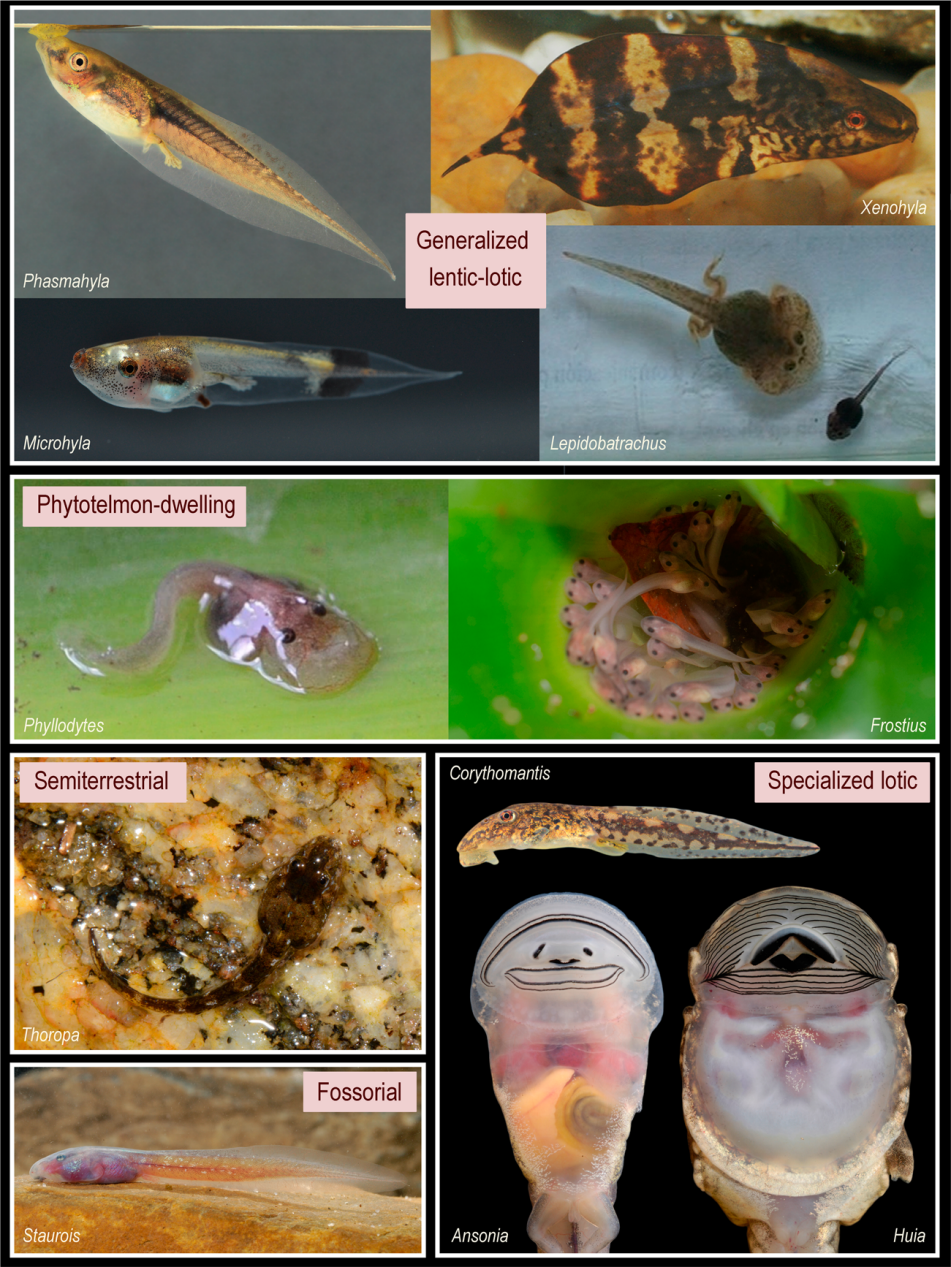
A variety of tadpoles assigned to different ecomorphological guilds. Classification based on McDiarmid and Altig^10^; see guild definition in “Methods”. In the category generalized lentic-lotic tadpoles, we join a wide larval ecomorphological diversity, including neustonic *Phasmahyla spectabilis* and nektonic *Xenohyla truncata* (Hylidae), suspension-feeding *Microhyla malang* (Microhylidae), and carnivorous *Lepidobatrachus laevis* (Ceratophryidae, stalking on *Pleurodema* tadpole). Among terrestrial tadpoles (i.e., inhabiting outside main water bodies) we include semiterrestrial tadpoles like *Thoropa taophora* (Cycloramphidae) that inhabit the spray zone of streams and waterfalls and phytotelmon-dwelling tadpoles such as *Phyllodytes gyrinaethes* (Hylidae); some endotrophic tadpoles such as *Frostius pernambucensis* (Bufonidae) also develop in water-filled bromeliad axils. Specialized lotic tadpoles inhabit water bodies with fast current and may develop large mouthparts (e.g., as in suctorial hylid *Corythomantis greeningi* and bufonid *Ansonia hanitschi)* and an abdominal sucker (e.g., in gastromyzophorous ranid *Huia cavitympanum)* to adhere to substrates. Finally, fossorial tadpoles such as *Staurois natator* (Ranidae) inhabit also lotic systems but they can bury in the substrate and avoid the current. We deeply thank photographs by M. Dubeux (*Frostius*), A. Haas (*Staurois*, *Ansonia*, *Huia*, *Microhyla*), F. Nascimento (*Phyllodytes*), and T. Pezzuti (*Phasmahyla*, *Xenohyla*, *Corythomantis*).

Tadpoles are a conspicuous part of the fauna that surrounds us. However, they were scarcely reported in traditional (i.e., morphology-based) taxonomy and virtually never included in the original description of anuran species (e.g., see the chronology of tadpole research in ref. ^10^). Identifications of subsequently described tadpoles were often hazardous, especially in species-rich regions, and often only based on the presence of adult specimens in the area. Innovating taxonomical practices such as molecular taxonomy and DNA barcoding in particular overcame the assignation impediment of tadpoles, boosted tadpole descriptions^11,12^, and enhanced the interest in this development stage. Far from a generalized idea (slippery things wriggling in muddy ponds), tadpoles have evolved an extraordinary diversity of shape (including unique adaptive characters), size, microhabitat use, and behavior, and this diversity may occur at higher taxonomic levels but also within smaller clades such as genera or species groups^13^. In this context, ecomorphological guilds were proposed to synthesize morphological diversity and emphasize the occurrence of similar trait sets in independent lineages. The most widely used scheme is that by Altig and Johnston^14^ (updated in ref. ^10^), which combines a heterogeneous set of characters (developmental modes, microhabitat, feeding behavior, and larval external morphology) to produce 21 ecomorphological categories (e.g., Fig. 1). Developmental mode is the first discriminator and distinguishes those tadpoles that feed actively on external sources (exotrophic) from those that develop exclusively from parental sources (endotrophic). Exotrophic tadpoles are divided following the type of water body they inhabit (lentic, lotic, or both) and then categorized according to microhabitat and behavior (e.g., benthic, arboreal, semiterrestrial) and trophic specializations (e.g., carnivorous, suspension feeder). Endotrophic species are classified into six groups that vary in the source of nutrition, the site of development, and the morphology of embryos and tadpoles (e.g., viviparous, direct developers). By definition, some of these latter species lack a tadpole, i.e., a “free-living larval stage” (although many typically larval characters and even a cryptic metamorphosis can be retained^15^), so premetamorphic stages are often referred to as embryos. The resulting 21 guilds provide a valuable tool to begin to explore larval ecomorphological diversity and its taxonomic and geographic distribution, and at the same time, may help to interpret patterns of knowledge gaps in large-scale analyses.

At first glance, the imbalanced knowledge of different life stages in anurans may have several underlying causes. Biological and ecological reasons would account for intrinsic features related to microhabitat use or development. For instance, many tadpoles inhabit ponds or pools and are relatively easy to collect, but several species develop in hidden, hard-to-access microhabitats. Some species have very short larval periods, and as mentioned, many frogs do not develop from free-living tadpoles. Geographical factors may involve remote areas with difficult access, and hotspots with an extraordinary, underestimated biodiversity, among others. Furthermore, historical and epistemological reasons may be related to the behavior of researchers, patterns of colonization and inventorying^1^, or access to regions with profound social conflict.

From all the above, the main purposes of this work are to estimate the extent of knowledge gaps regarding the diversity of tadpoles, interpret their biological and geographical patterns, and discuss possible causes and implications for other large-scale analyses. By framing our work in the theoretical context of shortfalls of biodiversity knowledge^1,4^, we expect to provide the first comprehensive assessment of our current level of ignorance on anuran tadpole biology.

## Results

### Described tadpoles per family and ecoregion

**Fig. 2:**
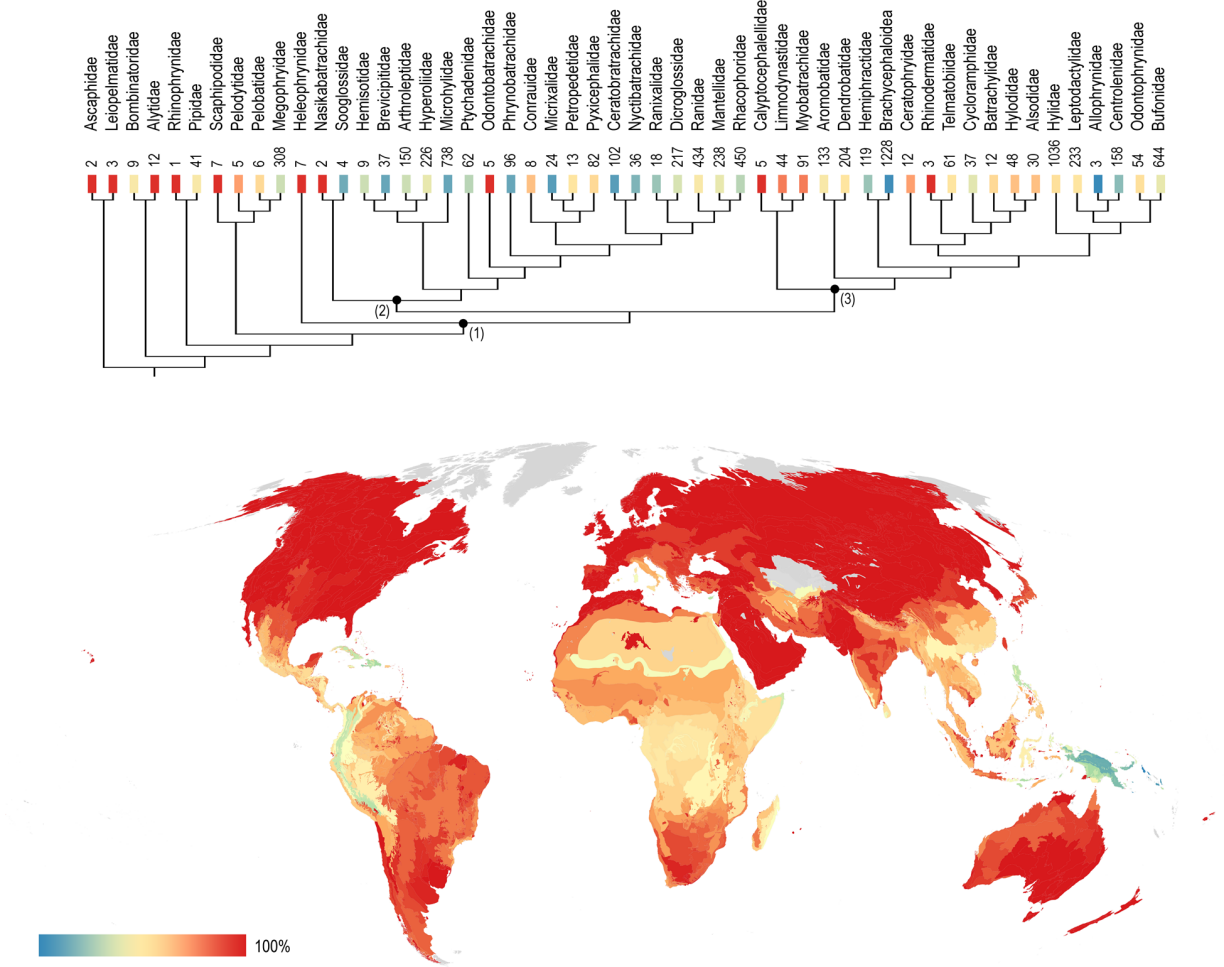
Taxonomic and geographic distribution of knowledge on anuran tadpoles. Phylogenetic hypothesis after Jetz and Pyron^64^. Colored squares below each terminal name indicate the percentage of tadpoles described as in the scale at the bottom left, and numbers summarize the species content; labeled nodes on the tree show the clades mentioned in the text: (1) Neobatrachia, (2) Indianura, (3) Notogaeanura. The map displays percentages of species with described tadpoles per ecoregion. Note the uneven distribution of knowledge, which is concentrated in scarcely diverse families and in the Holarctic region, and scarcer in most families of Indianura and in geographic regions of the Tropical Andes and New Guinea.

Our results show that 3088 out of 7507 species of frogs (41%) have larval stages described (Supplementary Table). Figure 2 illustrates percentages of descriptions in families and world ecoregions. At a taxonomic level (Fig. 2 top), 19 clades have more than 50% unknown larval diversity, and 68 out of 461 recognized genera (15%) have no tadpoles described to date. Although with a wide dispersion, most speciose families generally have lesser percentages described (see Supplementary Fig. 1), except for the highly diverse Hylidae (1036 species) with 65% of species with tadpoles characterized. Families with the highest (100%) and lowest (below 10%) percentages of tadpole descriptions are among the least speciose (e.g., Ascaphidae 2 spp., 100%; Allophrynidae 3 spp., 0%). Brachycephaloidea stands out as a highly diverse group (1228 spp.) with only 3% of premetamorphic stages described. In general, the tadpoles from non-neobatrachian families are fairly known. In Neobatrachia, the clade Indianura has mid-to-low percentages of description, except the small families Nasikabatrachidae, Odontobatrachidae, and Conrauidae with high values. Values are overall higher in Notogaeanura, with most species having tadpoles described among australobatrachians (Calyptocephalellidae, Limnodynastidae, and Myobatrachidae), Ceratophryidae, and Rhinodermatidae. Allophrynidae and brachycephaloid Ceuthomantidae are the sole families with premetamorphic stages undescribed to date.

Distributional data (Fig. 2 bottom) show that areas with ca. 100% tadpoles described concentrate in the northern hemisphere and some areas of the southern hemisphere, such as southeast Australia, Tasmania and New Zealand, and the Uruguayan savannas of South America. Those areas with lower percentages are the Northwest and Central Andes, Greater Antilles, most of New Guinea and New Britain-New Ireland islands, some Indonesian islands (Lesser Sunda and Sumba deciduous forest), the northern part of Filipinas (Luzon rainforest), and in Europe, Cyprus. Africa generally has lower description percentages than other regions, with the smallest values in East Saharan and Somali xeric woodlands. Northern New Guinea montane rainforest and the Solomon Islands are the ecoregions with the lowest proportion of tadpoles described (below 15%).

### Taxonomic and geographic distribution of larval exotrophic ecomorphological guilds

**Fig. 3:**
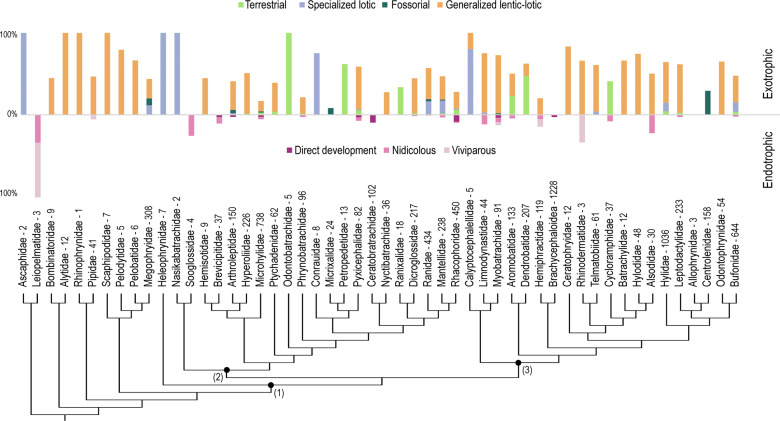
Taxonomic distribution of ecomorphological guilds of described tadpoles. Percentages of exotrophic and endotrophic guilds are shown above and below the 0 line. Numbers next to terminal names indicate species content, and labeled nodes on the tree show the clades mentioned in the text: (1) Neobatrachia, (2) Indianura, (3) Notogaeanura. Note the predominance of generalized lentic-lotic tadpoles, the restricted distribution of fossorial larvae, and the widespread occurrence of endotrophic developmental modes across the tree.

Among the 3088 species with premetamorphic stages described, 69% have generalized lentic-lotic tadpoles and 12% specialized lotic tadpoles, while terrestrial and fossorial guilds accumulate 9% and 3%, respectively. Taxonomic distribution of guilds (Fig. 3) shows that generalized lentic-lotic tadpoles are the single guild described in 12 families and predominate in other 22. Specialized lotic tadpoles occur exclusively in four families, including the earliest diverging anuran Ascaphidae, and neobatrachian Heleophrynidae, Nasikabatrachidae, and Conrauidae; these tadpoles also develop in other 11 families, but only predominate in Calyptocephalellidae. Some type of terrestrial development occurs in 17 families, being unique or majoritarian in five of them (e.g., Odontobatrachidae and Petropedetidae). In turn, fossorial tadpoles are described in eight distant families and are likely the single morph in Micrixalidae and Centrolenidae.

**Fig. 4:**
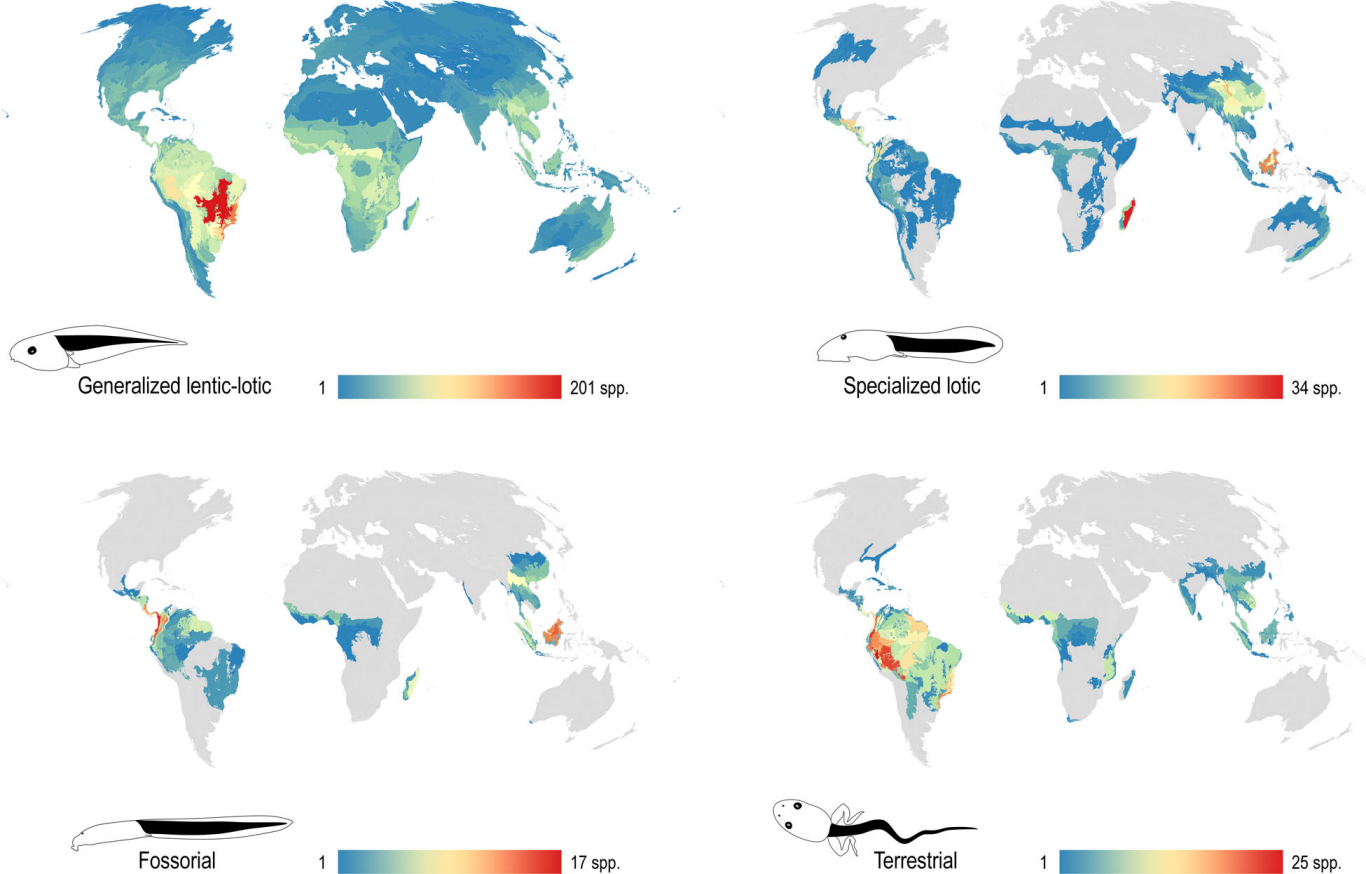
Geographic distribution of exotrophic guilds. Alpha diversity (species number per ecoregion) is indicated with colors as in the scales below each map. Note the worldwide distribution of generalized lentic-lotic larvae and the concentration of described tadpoles in several hotspots of anuran diversity, such as the Brazilian Cerrado, Tropical Andes, and the islands of Borneo and Madagascar.

The geographic distribution of larval exotrophic guilds is illustrated in Fig. 4. Generalized lentic-lotic tadpoles (1917 species) are distributed worldwide, with the highest alpha diversity in the Brazilian Cerrado (201 species), Serra do Mar coastal forest (168 species), and Bahia interior forest (167 species). Specialized lotic tadpoles (336 species) concentrate in Madagascar lowland (humid) and subhumid forests (34 and 33 species), and with lower values in Borneo lowland rainforest (28 species), Qionglai-Minshan conifer forest in China (25 species), and Petén-Veracruz moist forest in America (22 species). Fossorial and terrestrial tadpoles are described from similar areas (e.g., mostly tropical, west to the Wallace line). Fossorial tadpoles (100 species) show highest alpha diversity in the South American Chocó-Darién (17 species) and nearby areas of Northwest Andean forests (15 species), Magdalena Valley montane forest (15 species), and Isthmian-Atlantic moist forests (14 species), and in Borneo rainforests (14 species). In turn, terrestrial tadpoles (249 species) have the highest alpha diversity values in the Northwest Andean and Ucayali forests (25 species each).

### Ignorance about endotrophic ecomorphological guilds

**Fig. 5:**
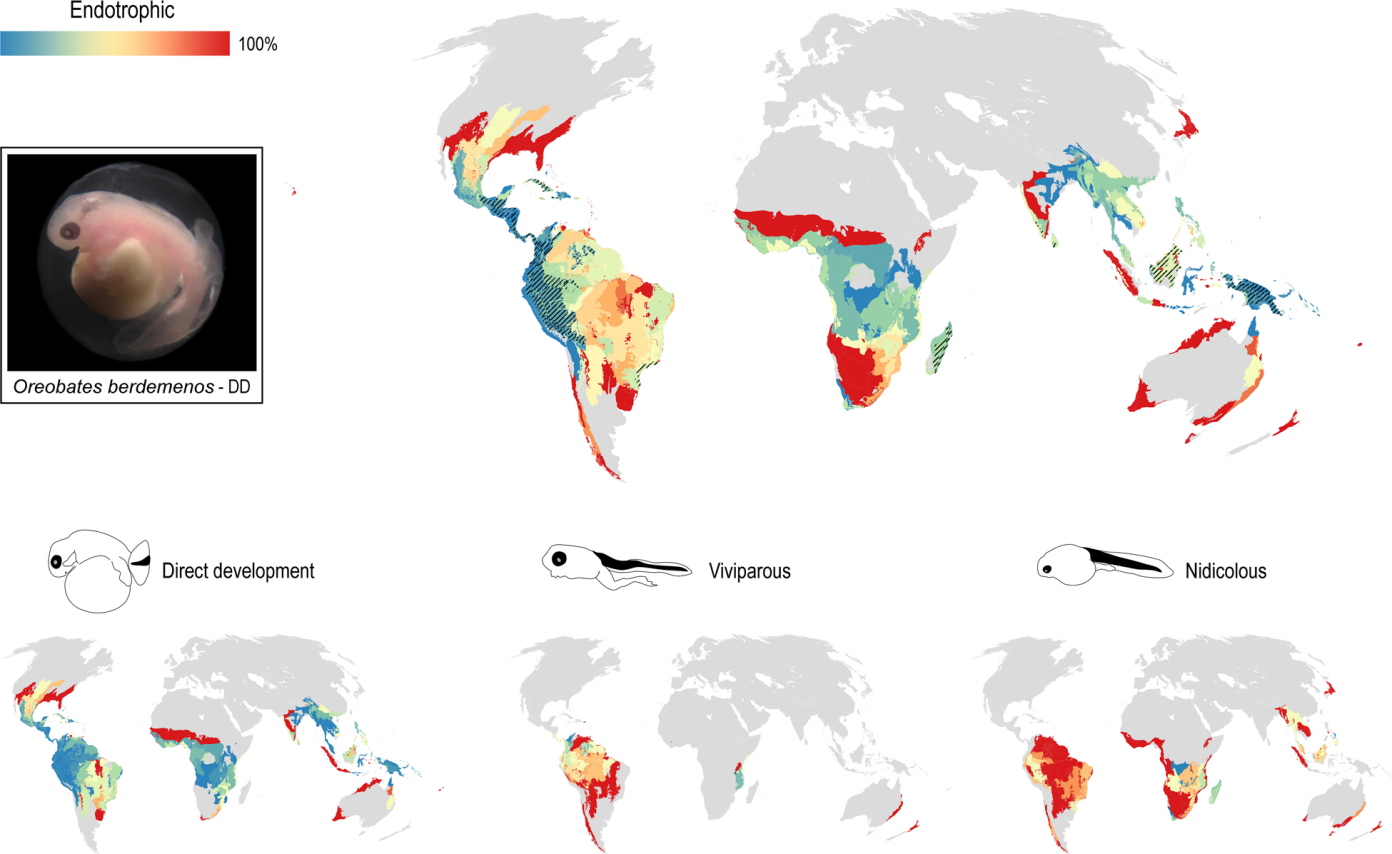
Geographic distribution of endotrophic guilds. The map at the top shows all endotrophic guilds together and includes inferred guild assignations (see text); it highlights the percentages of embryo/tadpole descriptions per ecoregion in colors following the scale and the areas with the highest alpha diversity of endotrophic species (>25 spp.; lined pattern). Note the overlap between areas with the highest diversity of endotrophic species and areas with minimal percentages of embryo/tadpole descriptions (Tropical Andes and New Guinea). The smaller maps at the bottom show the distribution and knowledge gaps of species with direct, viviparous, and nidicolous development. Note the high overlap of the three guilds in most ecoregions of South America, with an unbalanced knowledge in favor of nidicolous and viviparous tadpoles and the exclusive occurrence of some guilds in punctual regions. Direct-developing embryo of *Oreobates berdemenos* photographed by M. J. Salica.

Embryos and tadpoles with some type of endotrophic development are described for 226 (3%) of the 7507 anuran species. They are present in 23 taxonomic groups, being the single developmental type known in five of them (Fig. 3). However, at least 2303 species are known or suspected to have endotrophic nutrition (Supplementary Table), comprising about 30% of the anuran fauna. Among the 68 genera with unknown tadpoles, around 60% are likely endotrophic. The areas with the highest alpha diversity of species with known or suspected endotrophic development (>25 spp.) concentrate in South and Central America, Madagascar, Southwest India and Sri Lanka, and Borneo and New Guinea islands; however, these areas are those with the lowest percentages of embryos/tadpoles described (Fig. 5). Our estimation shows that in 398 ecoregions where endotrophic anurans are known or suspected to occur, less than a quarter of species have premetamorphic stages described. Among these, 132 (33%) show a total degree of ignorance (i.e., a presumed occurrence of that guild but no premetamorphic stage formally described).

Endotrophic guilds vary in their taxonomic and geographic distributions and the percentages of embryos/tadpoles described (Figs. 3 and 5). Direct development has been described in 112 species from nine clades but is also suspected in at least 1821 species plus two other clades (Petropedetidae and Sooglossidae). This accounts for approximately a quarter of all frogs, rendering direct development the most frequent endotrophic guild. Direct-developing species have wide distribution and are the single endotrophic guild in most of Central and North America, India, east of Wallace line, and northern regions of Australia. They explain exclusively the high diversity of endotrophic embryos in New Guinea, which remains mostly undescribed. In turn, nidicolous tadpoles are reported or presumed to be present in 18 families, in at least 156 species aside from the 87 where tadpoles are described. This accounts for 243 species, thus ca. 3% of frogs develop non-feeding tadpoles. The distribution of nidicolous tadpoles partly overlaps with that of direct-developing species, but it also reaches the southern Andes, Madagascar, Japan and New Zealand. The highest number of described tadpoles is in Malagasy’s humid and subhumid forests (ca. 12 species each), but more than half of the likely nidicolous larval diversity in these areas still needs to be discovered. Finally, some form of viviparity is reported or suspected in 7 clades (69 species, 1% of anurans), with the highest diversity in the northern regions of South America. Description percentages of these embryos are mid to low.

The threshold to dismiss commission errors of polygons ranges to species distributions did not reveal a significant effect at this level of analysis, and it also entails some artifacts in results (see Supplementary Figs. 2–4). For instance, it introduces certain noticeable omission errors, possibly due to the overlapping of small distributional ranges with ecoregions of varying extents, without accounting for transitional zones. As an example, it depicts no occurrences in Alaska when tadpoles of six species in that area are described. For these reasons, and while further exploratory analyses are pending, we opt for a parsimonious interpretation of distribution data as they were originally loaded and discuss main biogeographical patterns from maps accounting for species complete distributions.

## Discussion

Our study provides a comprehensive overview of the current knowledge of tadpole diversity, revealing significant gaps in our understanding. Surprisingly, despite the extensive research on different aspects of anuran biology, our findings demonstrate that more than half of the described species still lack information on their larval stages. Furthermore, current knowledge is highly uneven among taxonomic groups, ecomorphological guilds, and ecoregions. In the paragraphs below, we (1) summarize possible explanations for patterns of knowledge about tadpole diversity and (2) pinpoint some effects on other large-scale analyses regarding anuran biology and conservation. Finally, we comment on some aspects of the biphasic ontogeny in frogs in the context of shortfalls of biodiversity knowledge.

As expected, ecomorphological, biogeographical, and historical-epistemological aspects may explain general patterns of knowledge about tadpole diversity. First, intrinsic aspects of tadpole biology constrain the extent of knowledge of tadpole diversity. Description percentages generally decrease in lineages with a higher proportion of species known or suspected to have endotrophic development. Three groups hypothesized to be entirely endotrophic (Brachycephaloidea, Ceratobatrachidae, Brevicipitidae) bear the lowest indices of embryo descriptions (below 10%). Although most tadpoles described in the speciose family Microhylidae are conspicuous suspension-feeders^16^ (e.g., *Microhyla* in Fig. 1), the low description percentage in the clade can be explained by the high proportion of species presumably endotrophic (about 65%). The distribution of cophyline microhylids restricted to Madagascar explains the high richness of known and suspected nidicolous tadpoles on the island. Likewise, although most tadpoles described in African Arthroleptidae and Afro-Asian Rhacophoridae are lentic-lotic, significant portions of the diversity in both families (ca. 30 and 45% respectively) likely develop nidicolous or direct-developing embryos. The relationship between endotrophic development and the lack of knowledge of tadpole diversity is clearly evident in geographic data, where zones of highest ignorance coincide with the highest diversity of endotrophic guilds (compare maps in Figs. 2 and 5). Both the Tropical Andes and New Guinea, with the lowest percentages of tadpole description, house a high diversity of direct-developing and viviparous species whose ontogeny is unknown (e.g., Strabomantidae and Hemiphractidae in the Andes and a wide variety of direct-developing Ceratobatrachidae and asterophryine Microhylidae in New Guinea).

Taxonomic and geographic distribution of exotrophic tadpoles also contributes to interpreting patterns of knowledge on tadpole biology. Most families with more than 50% of their tadpoles described develop exclusive or predominantly generalized lentic-lotic tadpoles (e.g., Alytidae, Myobatrachidae). What we coded as generalized lentic-lotic tadpoles in fact includes a wide diversity in microhabitat use and trophic ecology. Although a thorough analysis is pending, these differences apparently do not affect the detectability of tadpoles and the percentages of tadpole descriptions. For example, high percentages are described in Ceratophryidae (with mostly carnivorous tadpoles^17^; e.g., *Lepidobatrachus* in Fig. 1), Pipidae (with mostly suspension-feeding tadpoles^18^) or in some megophryine genera (e.g., *Boulenophrys*, *Brachytarsophrys*; with exclusively neustonic tadpoles^19^). Generalized lentic-lotic tadpoles are almost the sole known guild in the Holarctic region, and this, along with the broad species distributions and the low diversity in that area^20,21^, would explain in part the high percentages of descriptions.

Several families that develop specialized lotic tadpoles exclusively or in the majority are in general well-known, probably because they are scarcely diverse (2–8 species, e.g., Ascaphidae, Conrauidae) and tadpoles are large and conspicuous, with large, sucker-like mouthparts^22^. Also, a comparatively low diversity in the environments where these tadpoles occur could render identification easier than in, for example, ponds where the number of similar syntopic species is usually higher. Almost a quarter of Bufonidae likely develops specialized lotic tadpoles (e.g., suctorial *Ansonia* in Fig. 1, gastromyzophorous *Atelopus*^23,24^), and about half of these are already described. Similarly, ca. 40% of Ranidae likely develop lotic tadpoles (e.g., ref. ^25^, gastromyzophorous *Huia* in Fig. 1), of which 40% are known. The geographic distribution of specialized lotic tadpoles, concentrated so far in Madagascar (Mantellidae) and Borneo (e.g., Bufonidae and Ranidae), contributes to the general mid-values of description percentages in both regions.

Terrestrial guilds have a taxonomic pattern of knowledge similar to that of lotic specialized tadpoles. Groups with higher description percentages are small families where terrestrial tadpoles develop exclusively or predominantly (e.g., African Odontobatrachidae—all five species described—and Petropedetidae—8 out of 13 species described). In Neotropical Aromobatidae and Dendrobatidae, the high proportion of phoretic species could increase the detectability of tadpoles in nature and contribute to the mid-high percentages of description in both families and their distribution zones. Tadpoles of at least 13 families develop in micro-waterbodies contained in plants^26^ (e.g., *Phyllodytes* and *Frostius* in Fig. 1). Although this could appear a priori as a hard-to sample microhabitat, these phytotelmon-dwelling tadpoles represent the highest proportion of terrestrial tadpoles described (unpublished data). As with specialized lotic tadpoles, the low diversity in these environments may balance a potentially lower detectability with a straightforward identification of tadpoles. Lastly, semiterrestrial tadpoles likely predominate in Cycloramphidae^27^ (e.g., *Thoropa* in Fig. 1) and almost half of them are already described. The restricted distribution of this family in the Brazilian Atlantic rainforest may explain the comparatively high description percentages in that region.

Regarding fossorial tadpoles, their microhabitat preferences and behavior of hiding or burying in the substrate could explain the overall lack of knowledge about this guild. The Centrolenidae is the best-known group, with 30% of their apparently exclusively fossorial tadpoles described in Chocó-Darién and near montane forests. In other families, several genera suspected to have only this type of tadpoles (e.g., arthroleptid *Cardioglossa* in Central Africa and megophryid *Leptobrachella* in East Asia^28,29^) have also about a third of their larval diversity described. Conversely, the tadpoles of only two species are described in the monotypic family Micrixalidae, endemic to the Western Ghats in India. Furthermore, micrixalid tadpoles remained unknown for more than a century (the type species was described in 1882) until 2016, when the first larvae were unearthed^30^.

Hortal et al.^1^ highlight that bias in biodiversity data toward specific regions, habitats, and environments involves syndromes related to historical patterns of colonization and inventorying and also to the behavior of researchers. This crucial issue was addressed in several analyses at a global scale, dealing with different taxonomic groups in biological and paleontological research^31,32^. In our specific case, a thorough analysis is in order but some main patterns can be easily interpreted. For instance, although tadpoles were described along with the first formal descriptions of frog species (e.g., *Pseudis* in ref. ^33^), the first specific works of larval morphology occurred during the nineteen century in Europe and North America^34,35^. This pioneer research, which surveys tadpole morphology of several typically Nearctic and Palearctic frogs (e.g., Alytidae, Pelobatidae, Scaphiopodidae), may also account for the high percentage of descriptions in those areas. High values in regions such as some South American grasslands and Indian plains and hills may be related to early local inventories of tadpole diversity^36–38^.

Regarding researcher behavior, we identify at least three main aspects that could explain the imbalanced knowledge of larval anurans. First, well-established schools of herpetological research working historically on some areas or taxa may explain why some taxonomic groups are better known than others. For instance, the high proportion of tadpoles described in Hylidae, the most diverse family among anurans, may be explained by its cosmopolitan distribution and accessibility for different research groups worldwide^39–42^. Second, specialization within the discipline (there are tadpole experts, tadpole literature, even tadpole scientific meetings^43^) can explain variation in description percentages among endotrophic guilds, ca. six times lesser in direct-developing species than in viviparous and nidicolous tadpoles. Unlike these two latter, which in general can still be characterized following standard procedures as in exotrophic tadpoles (e.g., using the developmental table in ref. ^44^), collecting, raising, and describing direct-developing embryos with a drastically different bauplan implies at least an out-of-routine task for tadpole specialists. Third, some methodological trends in taxonomic research, such as species descriptions based on molecular data, may have contributed to amplifying the gap between species descriptions and tadpole descriptions. In fact, a quick plotting of the two latter along the years shows that curves highly diverge from yr. 2000 onward, after which species discrimination by molecules produced a noticeable speed-up in species descriptions (Supplementary Fig. 5). The recent inclusion of tadpole characterization in species descriptions (with unambiguous assignation by DNA barcoding^45^) and approaches through integrative taxonomy^46^ remain to be improved to compensate the boost of new species discovery.

The implications of missing information about the ontogeny of organisms with complex life cycles extend beyond what can be addressed in just a few paragraphs. Immediate consequences in several disciplines may be envisioned after comparing conclusions from large-scale analyses based on different life stages. This is crucial in the context of the global biodiversity crisis^47^ due to the assignation of taxa to threat categories and the definition of Priority Conservation Areas are strongly biased toward knowledge of the adult phase (e.g., see ref. ^5^). Given the vast extent of our knowledge gaps on tadpole biology and the current trends in habitat transformation, one of the most significant implications we can highlight is the potential extinction of complex organisms that have yet to be recognized by science. Several examples, many recently discovered, reveal an extraordinary ecomorphological diversity that cannot be adjusted in our present larval guild scheme (e.g., the unique gastromyzophorous, phytotelmon-dwelling larvae of *Phyllodytes gyrinaethes*^48,49^, or the exotrophic tadpoles of *Limnonectes larvaepartus* that develop initially within the mother oviducts^50^). The disappearance of further rare or even unknown ecomorphotypes is feasible. Although this would be a worldwide concern, effects could be more profound in many of the most important regions regarding anuran diversity and conservation.

In this regard, global hotspots for anuran diversity^21^ differ in the extent of knowledge about tadpole diversity. Among the richest areas, whereas the lowlands of Amazonia and the Brazilian Atlantic forest are reasonably well described, the Andean region of Amazonia stands out as markedly understudied. Land use intensification would have a decisive impact on local anurofauna^51,52^, specifically on the scarcely known terrestrial and endotrophic tadpoles concentrated in those areas. Habitat split, i.e., human-induced disconnection between habitats used by different life history stages of a species, and water pollution are identified as major threats for species with aquatic tadpoles in this ecoregion^53^. In the second place, recent studies revisit patterns of amphibian richness and the extraordinary amount of endemisms in Madagascar^54,55^. Larval diversity in this hotspot is relatively well-known mainly due to systematic prospecting in the last decades^56^. This diversity includes exceptional, highly divergent ecomorphotypes (e.g., sand-eating tadpoles^57^) and a high concentration of specialized lotic and nidicolous tadpoles. Given current habitat destruction and fragmentation threats, conservation efforts are crucial in Malagasy eastern rainforests that still harbor a significant portion of undescribed diversity^55^. Finally, although comparatively less studied, the African rainforest anuran fauna stands out with high richness and proportion of endemisms^58^. Tadpole diversity in Cameroonian-Congolian forests is described in about half, and among potential threats to conservation, specific effects on eggs and tadpoles are foreseen^59^.

On the other hand, assessing the vulnerability of anuran species while ignoring larval stages potentially susceptible to different threat drivers clearly represents a significant drawback and may lead to profound underestimation of extinction risks. It is concerning to note that tadpoles are entirely unknown for nearly half of the frogs categorized as Least Concern (unpublished data). This implies that the “Low” extinction risk has been estimated from a partial scenario, overlooking that threats faced by larval stages could indeed be a concrete cause of local or even total extinction. Paradoxically, anurans are the tetrapod group with the highest number of species assessed as Data Deficient^60^, which is one of the most significant challenges for accurate decision-making^61^. Considering the substantial number of species assessed without a tadpole description, we suggest that the number of DD species might be underestimated.

The need for morphological knowledge and characterization of premetamorphic stages of anurans significantly hampers progress in disciplines such as comparative anatomy, systematics, and evolution. Large-scale phylogenies of anurans incorporating morphological evidence have relied on larval characters as the primary source of phenotypic variation^62,63^. However, these studies have only included a relatively small number of representative species of the diversity of Anura. More recently, well-sampled phylogenies of the order were exclusively based on molecular information^64,65^, providing a relatively well-resolved framework of relationships among major lineages. Nevertheless, many nodes throughout the entire tree need to be better supported and lack phenotypic diagnosis (see ref. ^66^). Further efforts to fill the knowledge gaps regarding larval morphology in anurans will positively affect understanding the phylogenetic relationships and evolutionary patterns of many phenotypic traits.

To end with, biphasic ontogeny in anurans can be discussed in the context of shortfalls of biodiversity knowledge. These shortfalls are defined as the gaps between realized/extant knowledge within a biological domain at a given time and concern taxonomic (species identity), extrinsic (geographic distribution, population dynamics, evolutionary relationships) and intrinsic attributes of species (functional traits, abiotic niche, biotic interactions^1^). The recent definition of a new shortfall type, the Haeckelian shortfall by Faria et al.^4^, brought the unnoticed importance of ontogenetic development into discussion. This was imminent, especially because complex life cycles are prevalent among animal phyla^67^ and living organisms in general^68^. As stressed by Faria et al.^4^, relationships among the Haeckelian shortfall and the previously synthesized types exhibit two-way dynamics: knowledge on ontogenetic aspects contributes to filling gaps of knowledge in other dimensions (e.g., phenotypic characterization of semaphoronts helps to generate more comprehensive phylogenetic hypotheses) and at the same time, knowledge on different life stages must be nourished with specific information covered by the remaining shortfalls (e.g., intrinsic and extrinsic attributes may differ significantly along life stages). The latter is reflected in the hierarchical structure that Faria et al.^4^ give to the interrelation scheme among shortfall types.

In anurans, biological differences among larval and adult forms are astonishingly profound and concern not only morphology and physiology but also ecology and behavior, the very attributes that in adults merited the definition of specific shortfall types. To begin with, tadpoles in general have their description separated from that of the species (i.e., only a third of tadpole descriptions published in *Zootaxa* journal are part of studies describing new species^69^). Some anuran species have been described based on premetamorphic stages, always very conspicuous animals that are easily differentiated from other species (e.g., *Pseudis paradoxa*^33^; *Clinotarsus penelope*^70^; *Calyptocephalella gayi*, but see ref. ^71^ for a different interpretation), and numerous candidate species hypothesized from larval morphology await formal description (e.g., the strange tadpole of ref. ^37^ waited for almost 100 years to be assigned to the recently described adults of *Nasikabatrachus sahyadrensis*^72^). This makes ignorance about tadpoles as basic entities resemble a Linnean shortfall.

Excepting the Wallacean shortfall (we assume that knowledge about a species distribution based on adult forms does not differ from that inferred from its tadpoles), other shortfalls can be equally invoked. A Haeckelian-Prestonian shortfall relationship is interpreted since population dynamics in larval and adult phases may differ significantly. For instance, the spatial and temporal distribution of adults that breed during brief lapses of explosive reproduction while tadpoles remain in ponds^73^ may render very different scenarios in population ecology studies. Although evolutionary relationships are intrinsically the same for adults and tadpoles (disregarding that in practice hypotheses are contingent on datasets), the evolution of traits also covered by the Darwinian shortfall may reveal varied arrangements prior to and after metamorphosis. For instance, Sherratt et al.^74^ found rampant homoplasy linked to ecology in body shape evolution among tadpoles of Australian anurans, as opposed to the adult morphospace for which phylogenetic structure is stronger.

As to extrinsic attributes, given that in most anurans the metamorphosis implies a reset in the fundamental variables of the ecological niche (aquatic to terrestrial environment), aspects related to responses to abiotic conditions (Hutchinsonian shortfall), ecological functions (Raunkiæran shortfall) and biotic interactions (Eltonian shortfall) can be radically divergent in tadpoles and cannot be inferred from knowledge in the adult phase. For instance, Bolochio et al.^51^ addressed the first global-scale analysis for anuran conservation from a functional perspective. This study, framed in the Raunkiæran shortfall type, stands out as a clear example of how contrasting the categorization (i.e., adult ecomorphs differ considerably from larval ecomorphological guilds), interpretation, and eventual decision-making may be (e.g., the most vulnerable adult ecomorph does not include a high proportion of the most neglected larval guilds) when knowledge is grounded on tadpole vs. adult frog datasets.

### Final remarks

The results of our study underscore the pressing necessity for additional research on tadpole diversity. Whereas the data we provide contribute to filling knowledge gaps in aspects related to Haeckelian-Linnean (by quantifying tadpole descriptions over anuran species) and Haeckelian-Raunkiæran shortfalls (by analyzing taxonomic and geographic distribution of larval functional groups), many other aspects of larval biology remain underexplored, especially in a global context. Our initial dataset may serve as a basis for many future analyses that lay on more refined ecomorphological characterization^75^, ecomorphological evolution^76^, and quantification and comparison of patterns and determinants of knowledge shortfalls across vertebrate groups^77–79^. This kind of research is crucial not only for anuran conservation but also concerning its potential significant applications in various scientific disciplines.

## Methods

### Datasets

#### Species list and taxonomy

We worked with the species list, nomenclature, and taxonomic arrangement for Anura by Frost^80^ (as available in October 2022). This yields a dataset of 7507 species distributed into 51 families plus the superfamily Brachycephaloidea (with five families—Brachycephalidae, Ceuthomantidae, Craugastoridae, Eleutherodactylidae, Strabomantidae—and three genera not assigned to family).

#### Tadpole descriptions

For each 7507 species, we first consigned whether or not larval stages are known. We collected this information from varied sources, including the AmphibiaWeb and Amphibian Species of the World webpages^80,81^, manual Google searches, and our libraries and expertise. We considered published formal descriptions but also descriptions in comprehensive works such as phylogenetic analyses, keys, books, and available dissertations. We tried to check the most comprehensive and recent compilations (e.g., generic revisions, comparative studies) to avoid tadpole misidentification problems.

#### Larval ecomorphological guilds

We assigned each described tadpole to one of seven ecomorphological guilds summarized from the scheme by McDiarmid and Altig^10^ as follows:

(A) Exotrophic tadpoles:

1. generalized lentic-lotic: tadpoles that inhabit ponds or quiet zones of lotic systems. In this guild, we include a wide variety of ecomorphological guilds originally defined as benthic, nektonic, neustonic (with upturned mouthparts), macrophagous, carnivorous, suspension-feeding, and suspension-rasper tadpoles. For the sake of simplicity, we do not discriminate microhabitat or trophic ecology in this instance.
2. specialized lotic: stream tadpoles with morphological specializations for inhabiting water bodies with faster current. These include adherent, clasping, suctorial, and gastromyzophorous (with abdominal sucker) tadpoles as originally defined.
3. fossorial: stream tadpoles that live buried among substrate (sand, leaves, gravel), as originally defined. Psammonic tadpoles (bury in the sand) are a different ecomorphotype in the reference scheme, but here we include them among fossorial.
4. terrestrial: tadpoles that can be found outside main water bodies. These include phytotelmon-dwelling (inhabit water-filled tanks in plants) and semiterrestrial tadpoles (inhabit the spray zone in streams) as originally defined, plus tadpoles that develop in underground chambers or caves. We also include species whose adults transport tadpoles in their backs (phoretic) to highlight the out-of-water phase of their development.

(B) Endotrophic tadpoles/embryos:

(5) nidicolous: non-feeding tadpoles that develop in various places, including nests, ground cavities, phytotelma, ponds, etc.
(6) viviparous: embryos that develop within some part of the parent body. This includes viviparous, ovoviviparous, exoviviparous, and paraviviparous guilds as originally defined.
(7) direct developers: embryos that develop within terrestrial eggs before hatching as small froglets.

The last two guilds lack a tadpole, i.e., a “free-living larval stage”; in these cases, we considered descriptions of embryonic morphology and refer to them as embryos when possible.

#### Phylogeny

For analysis and discussion, we followed the phylogenetic hypothesis of Jetz and Pyron^64^, which incorporated nuclear and mitochondrial sequences for 3449 anurans, including representatives from all extant families.

#### Distribution data

Species distributions were obtained from the IUCN database^60^. We downloaded digital range maps (extent of occurrence maps) for 6235 anuran species available at this source. Given the large number of species, the global extent of the analyses, and the bias associated with the source of the distributional data^2^, we decided to run the analyses using ecoregions as a biogeographical unit^82^. In our opinion, maps based on these biological units are more realistic in a biological sense and easier to interpret than the “traditional” maps based on a grid in which biogeographical units could be mixed. Furthermore, using range maps at finer resolutions would increase the biases related to overinterpreting the limited information contained in these maps^83,84^.

### Analyses

#### Tadpole descriptions in a taxonomic context

We generated a first dataset containing 7507 anuran species, indicating for each its familial/suprafamilial level, whether or not tadpoles are described, and the ecomorphological guild these are assigned to (Supplementary Table). In a few species, both exotrophic and endotrophic tadpoles have been described (e.g., *Incilius periglenes*); to simplify, facultative conditions were coded prioritizing the exotrophic guild. We then calculated and plotted percentages of species with tadpoles described per family and guild diversity among described tadpoles.

#### Mapping general ignorance and guild diversity

We explored our general ignorance about tadpole description and distribution of larval ecomorphological guilds worldwide. First, using the “join by location function” of QGIS^85^ we performed a geographic match between the geographic distribution of each species and its recompiled developmental information. After the match, we recovered a database encompassing 6235 species (Supplementary Table), for which both kinds of information are available. Using QGIS [“join attributed by location (summary)”] we overlapped the terrestrial ecoregions of the world with the species attributes, and we counted the number of species per ecoregion to generate the following maps and estimations:

1. the percentages of species with tadpoles described per ecoregion (considering the total number of the species in our database), and
2. the species richness of each ecomorphological guild per ecoregion. In this case, we cannot estimate percentages because the total number of tadpoles of each guild per region is not available (it would require assigning guilds to unknown tadpoles; but see next section). Thus we filtered and split the former database generating a new sub-database per guild (a total of 2812 species) containing only those species with already described tadpoles. By repeating the above-described procedure we could estimate and map the alpha diversity of each guild in each terrestrial ecoregion of the world.

To explore the effect of commission errors arising from inaccuracies in polygon distributions, we employed a threshold approach. Specifically, for a given species we only included those ecoregions overlapping with a minimum of 5% of its total range. To achieve this, we first utilized QGIS to perform an intersection analysis, overlaying the species distribution data with the vector file representing ecoregions. This allowed us to calculate the percentage of each species distribution contained within each individual ecoregion. Subsequently, we excluded those portions of the species range overlapping with “marginal ecoregions” (i.e., below the 5% threshold of their entire distribution).

#### Ignorance about species with endotrophic development

We applied a different approach for three endotrophic guilds by estimating the total numbers of species per guild based on the literature. Thus, in addition to species with described endotrophic tadpoles, we included those species where the endotrophic guild is suspected based on reproductive biology aspects (e.g., oviposition site, size and number of eggs, etc.; Supplementary Table). Although endotrophic guild distribution appears to be highly conserved at several levels (e.g., all known ceratobatrachids are direct developers, and all known cophyline microhylids have nidicolous tadpoles^86,87^), variation is known even within genera (e.g., *Gastrotheca* includes tadpole-producing and viviparous species; *Philautus* includes species with nidicolous and direct-developing embryos^88,89^). We were conservative enough not to assign guilds in groups with significant variation, but we did generalize a single type in cases where a second guild is exceptional (e.g., only *Eleutherodactylus jasperi* is viviparous while remaining known brachycephaloids are known or suspected to be direct developers^90^). For those species where some form of endotrophic development is suspected but the specific type cannot be identified (e.g., some brevicipitid genera), we coded only endotrophic. Using this new dataset, we estimated the percentages of described tadpoles of each endotrophic guild per ecoregion.

Although with an unlikely profound impact, we acknowledge several potential sources of bias in our dataset, results, and interpretations. First, specific identification of tadpoles (i.e., assignation to a species and corresponding adult) is not minor and a prerequisite of any subsequent reliable description. Certainty in tadpole identification may vary among groups (e.g., it may be easier in areas with low diversity or species with parental care) and times (e.g., molecular identification is becoming more frequent^11,91^). Especially, some old literature deals with widely distributed species that have been subsequently split into several taxa, and it is not always possible to know accurately to which one the original mention refers; in those cases, we retained the tadpole description as originally assigned. Similarly, some tadpoles mentioned in the first editions of books that are no more reported in the following editions are subject to doubt. Although we checked for comments on misidentification and synonymies during our search, we may have overlooked some examples. Second, the definition and assignation to ecomorphological guilds may differ from other schemes in use, which may render different interpretations compared to other analyses. For instance, the definition of direct development we apply differs from that in Liedtke et al.^76^, and thus some calculations along the anuran tree may vary. Finally, even after a threshold approach, the geographic data we depict may overestimate the distribution of some species and guilds. For instance, northern records of viviparous Darwin’s frog *Rhinoderma darwinii* extend artificially the distribution of the species and its ecomorphological guild into the whole ecoregion of Chilean Matorral.

### Supplementary information

Supplementary Figures

Supplementary Table

## Data Availability

All data generated or analyzed during this study are included in this published article and its supplementary information files.
