# Supplementary material for: Global shortfalls of knowledge on anuran tadpoles
Source: NPJ Biodivers. 2023 Oct 30;2:22. doi: 10.1038/s44185-023-00027-1 (PMC11332183; doi:10.1038/s44185-023-00027-1)
Supplement: Supplementary file 1 — Supplementary Figures [file 44185_2023_27_MOESM1_ESM.pdf]

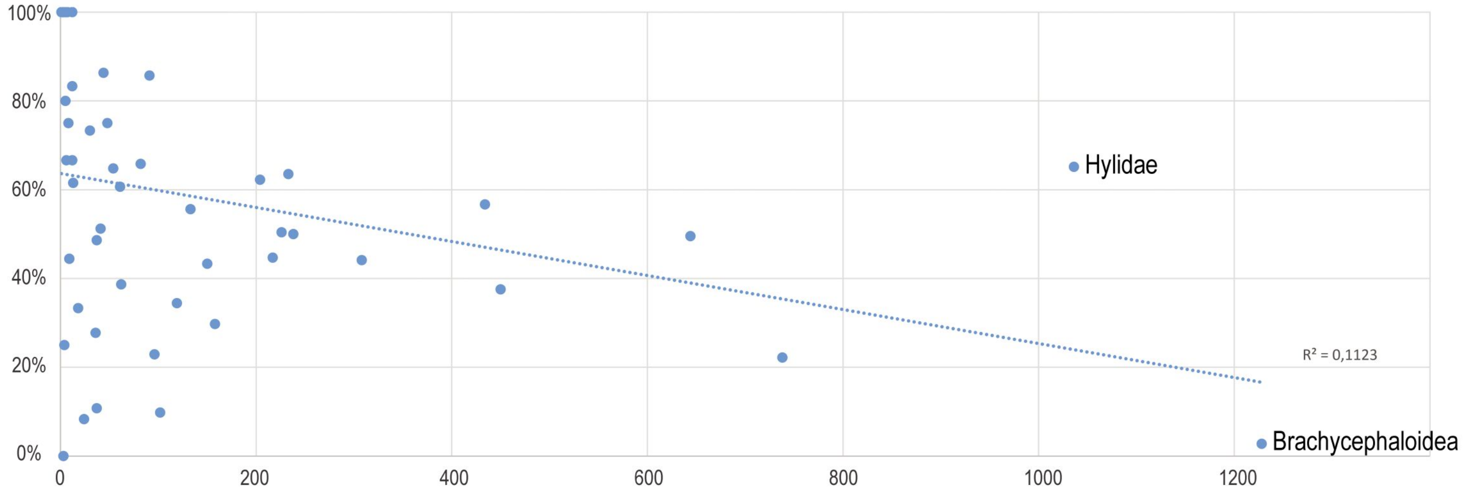

Supplementary Figure 1. Specific diversity and percentage of tadpole description per group. Note the general trend of more speciose clades to have lower description percentages, and the salient cases mentioned in text.

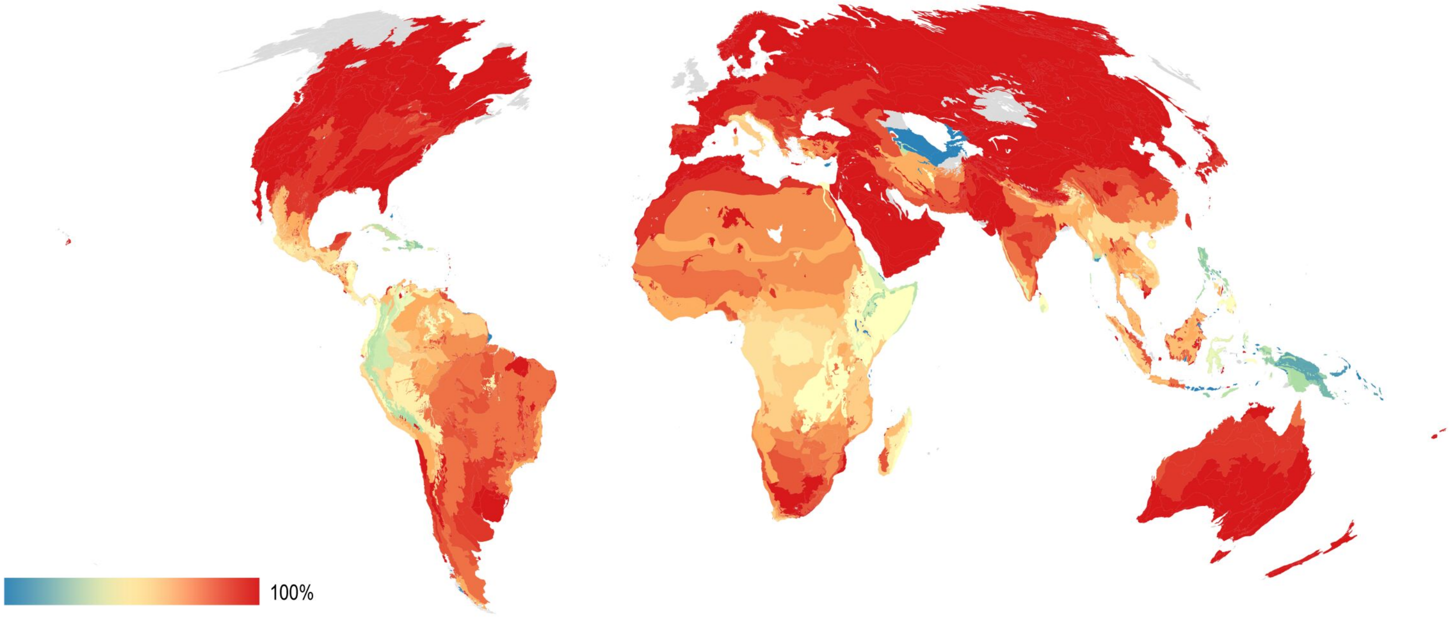

Supplementary Figure 2. Geographic distribution of knowledge on anuran tadpoles. Percentages of species with described tadpoles per ecoregion, after applying a threshold of 5% to species distributions.

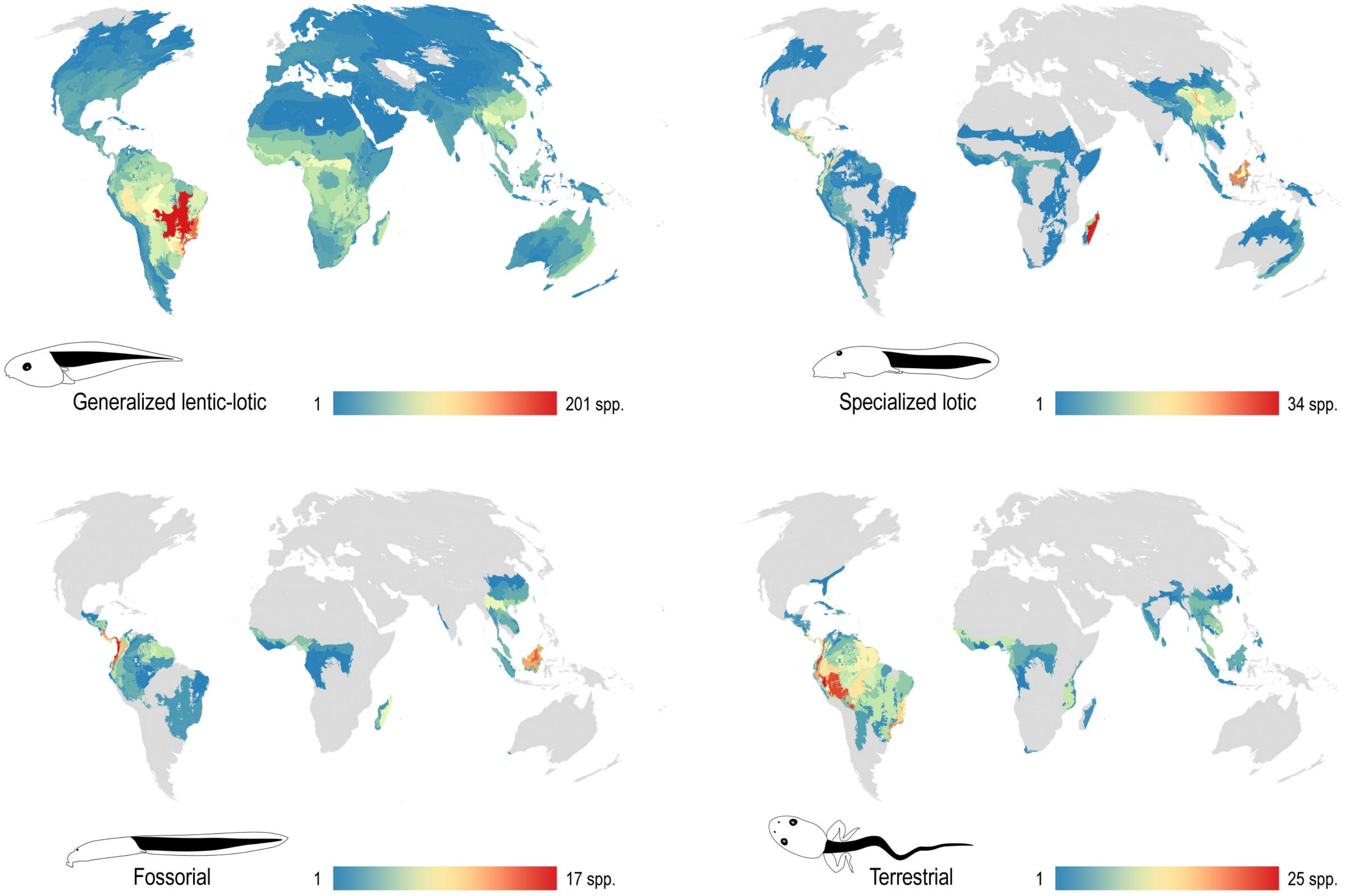

Supplementary Figure 3. Geographic distribution of exotrophic guilds, after applying a threshold of 5% to species distributions.

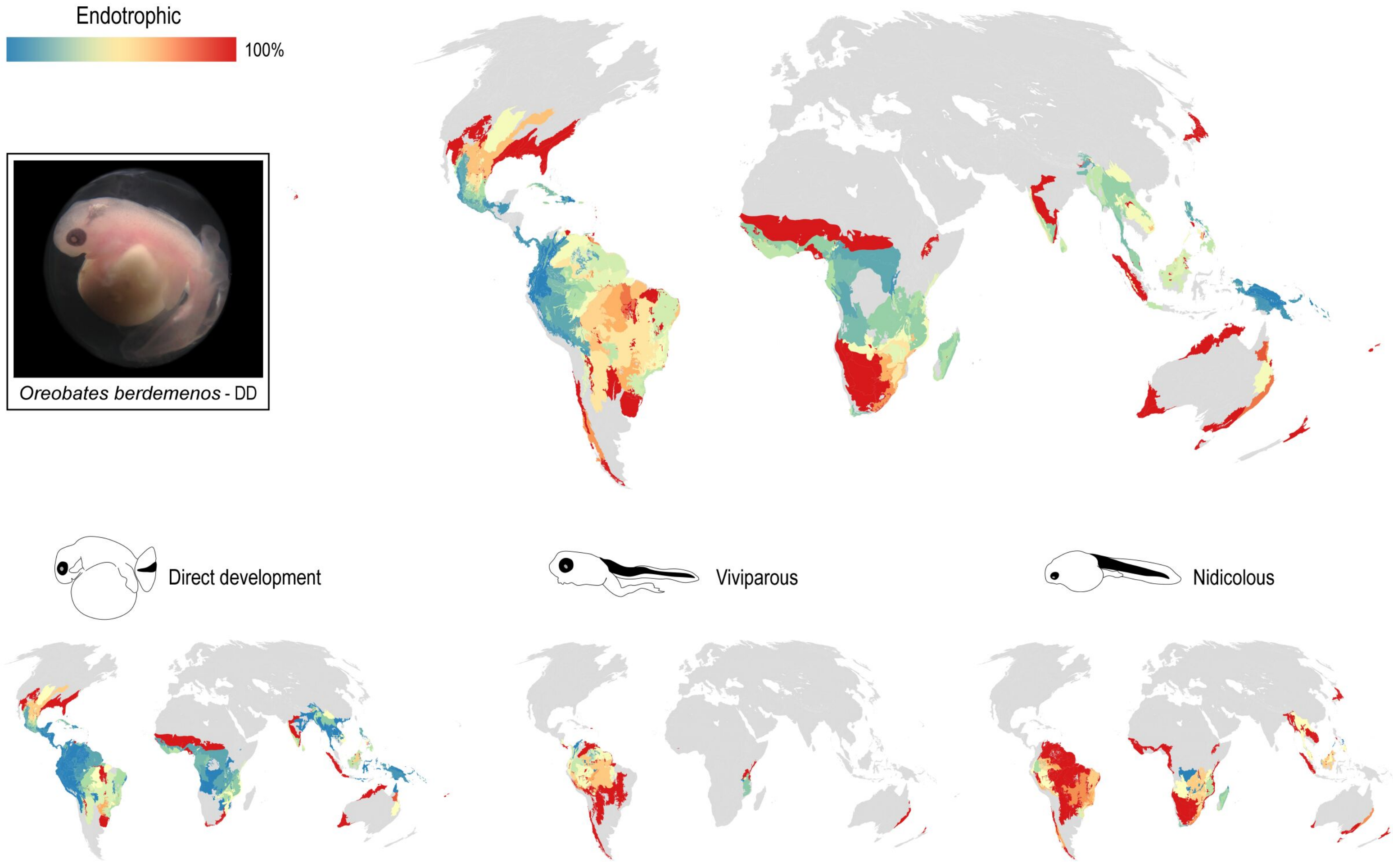

Supplementary Figure 4. Geographic distribution of endotrophic guilds, after applying a threshold of 5% to species distributions.

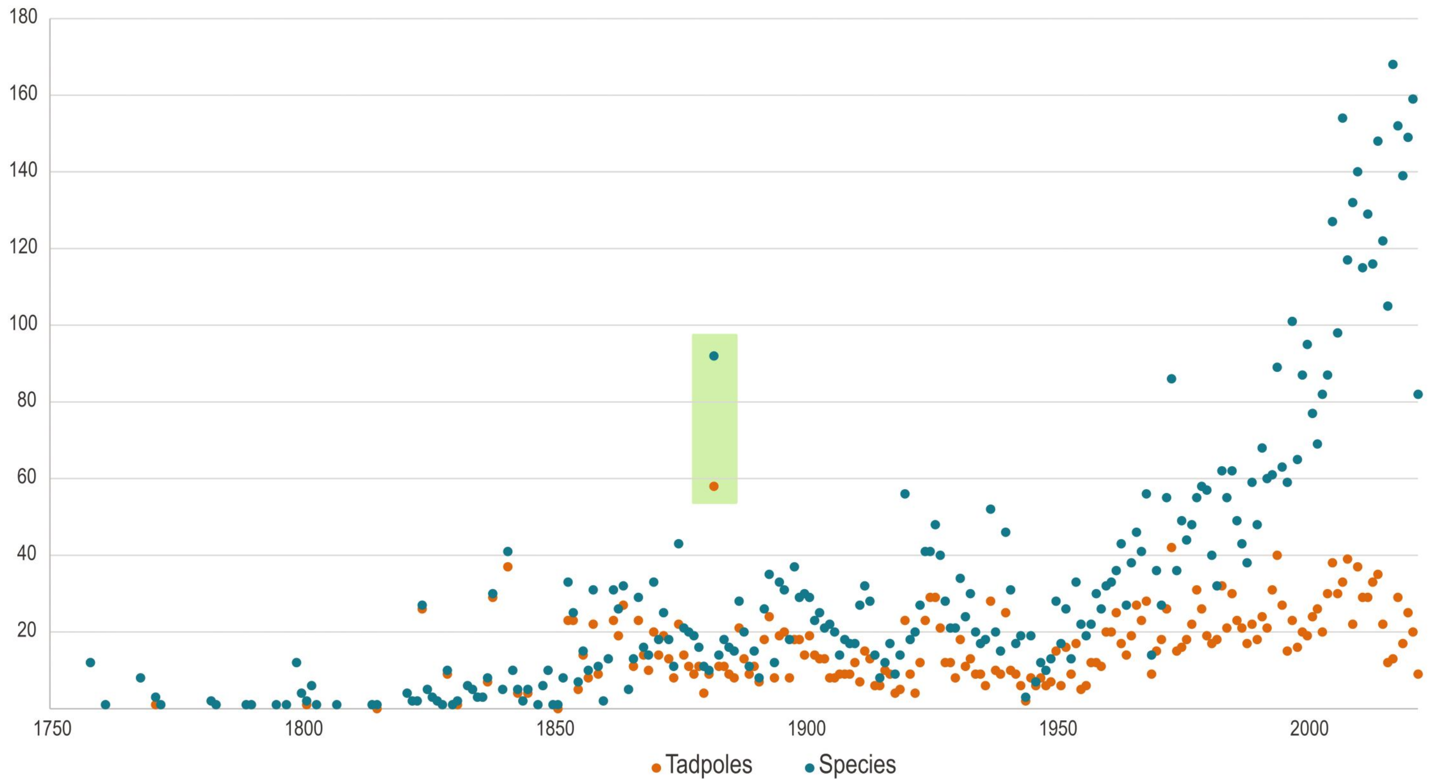

Supplementary Figure 5. Species and tadpole descriptions through the years. Note the high increase of species descriptions after 2000 yr. The square highlights the significant contribution by Boulenger 1882, ca. 40% of which still lacks tadpole descriptions.
